# Vascular Risk Factor Profiles Differ Between Magnetic Resonance Imaging-Defined Subtypes of Younger-Onset Lacunar Stroke

**DOI:** 10.1161/STROKEAHA.117.017813

**Published:** 2017-08-01

**Authors:** Loes C.A. Rutten-Jacobs, Hugh S. Markus

**Affiliations:** From the Department of Clinical Neurosciences, Stroke Research Group, University of Cambridge, United Kingdom.

**Keywords:** population control, risk factors, smoking, stroke, white matter

## Abstract

**Background and Purpose—:**

Differing associations of vascular risk factors with lacunar infarct have been reported, which is likely because of diagnostic differences and possible heterogeneity in the pathogenesis underlying lacunar infarction. In a large magnetic resonance imaging–verified cohort of lacunar infarct patients, we investigated the risk factor profile of lacunar infarction and magnetic resonance imaging characteristics.

**Methods—:**

One thousand twenty-three patients with lacunar infarction (mean age, 56.7; SD, 8.5) were recruited from 72 stroke centers throughout the United Kingdom as part of the UK Young Lacunar Stroke DNA Study. Risk factor profiles were compared with 1961 stroke-free population controls with similar age. Furthermore, we tested risk factor profiles of lacunar stroke patients for association with the presence of multiple lacunar infarcts, white matter hyperintensities (WMH), and location of the acute lacunar infarct.

**Results—:**

Hypertension (odds ratio [OR], 2.21; 95% confidence interval [CI], 1.85–2.64), diabetes mellitus (OR, 2.10; 95% CI, 1.61–2.73), hyperlipidemia (OR, 1.74; 95% CI, 1.46–2.07), and smoking (OR, 1.65; 95% CI, 1.39–1.96) were independently associated in lacunar infarct patients compared with healthy controls. Patients with multiple lacunar infarcts were more likely to be men (OR, 2.53; 95% CI, 1.81–3.53) and have hypertension (OR, 1.54; 95% CI, 1.12–2.04) compared with patients with a single lacunar infarct, independent of other vascular risk factors. The presence of moderate-to-severe WMH versus no or mild WMH was independently associated with increased age (OR, 1.54; 95% CI, 1.12–2.04), hypertension (OR, 2.06; 95% CI, 1.44–2.95), and impaired renal function (OR, 0.90; 95% CI, 0.82–0.98).

**Conclusions—:**

In this magnetic resonance imaging–verified lacunar stroke population, we identified a distinct risk factor profile in the group as a whole. However, there were differing risk factor profiles according to the presence of multiple lacunar infarcts and confluent WMH. The association of hypertension, smoking, and renal impairment with the presence of multiple lacunar infarcts and confluent WMH might reflect a diffuse small vessel arteriopathy.

## Abstract 2

Supplemental Digital Content is available in the text.

Lacunar stroke accounts for nearly a quarter of all ischemic strokes and is an important cause of vascular cognitive impairment and dementia.^1,2^

Despite its importance, the pathogenesis of lacunar stroke is less well understood than that of other stroke subtypes, such as large artery disease and cardioembolism. This is partly because lacunar stroke is rarely fatal during the acute phase, and, therefore, there is a paucity of pathological data. An additional reason is that lacunar infarcts are often not well seen on computed tomography, and accurate phenotyping requires magnetic resonance imaging (MRI). It has been shown that the computed tomography-based methods combined with a lack of detailed investigation of embolic sources as used in many epidemiological studies and clinical trials can overdiagnose small vessel disease; as many as 50% of cases diagnosed as lacunar stroke by such definitions turn out to be because of other causes (such as large artery disease) when more detailed phenotyping, including the use of MRI, is performed.^3^ However, there have been few epidemiological studies using MRI-based subtyping.^4–9^

The picture is further complicated by data suggesting the arterial pathology underlying lacunar stroke is heterogeneous. In the 1960s, C. Miller Fisher suggested there may be 2 main pathologies; focal microatheroma at the origins or proximal proportions of the larger (200–800 µm diameter) perforating arteries, which usually results in large isolated lacunar infarcts, and a more diffuse arteriopathy of the smaller vessels (40–200 µm diameter) resulting in multiple smaller lacunar infarcts.^10^ Subsequent imaging studies suggested these 2 subtypes could be differentiated on brain imaging, and multiple lacunar infarcts were often found to be associated with confluent white matter hyperintensities (WMH).^11^ Further studies suggested these 2 parallel radiological phenotypes, namely isolated lacunar infarcts associated with focal microatheroma and multiple lacunar infarcts/WMH associated with a diffuse small vessel arteriopathy. In addition, it has been hypothesized that small vessel disease of the brain might be part of a systemic small vessel arteriopathy.^12^ This is supported by numerous examples in literature of the occurrence of small vessel disease in the brain in patients with impaired kidney, retina, or heart function.^12^

One way of exploring differences between radiological variants of lacunar stroke and determining whether they are indeed distinct subtypes is comparing their risk factor profiles.

In this study, in a large cohort of younger-onset MRI-confirmed lacunar stroke, we determined the risk factor profiles for lacunar stroke as a whole and MRI-based subtypes. Lacunar stroke subtypes were determined according to number of lacunar infarcts and severity of WMH. In addition, we performed analysis of risk factor profiles stratified by infarct location because previous studies hypothesized that there might be heterogeneity in disease mechanism according to small vessel disease location.^13,14^

## Materials and Methods

### Study Population

A total of 1023 white patients with MRI-confirmed lacunar stroke, aged ≤70 years, were recruited from 72 specialist stroke centers throughout the United Kingdom, between 2002 and 2012, as part of the young lacunar stroke DNA resource (online-only Data Supplement).

The study was approved by the Multi-Center Research Ethics Committee for Scotland (04/MRE00/36), and informed consent was obtained from all participants.

Lacunar stroke was defined as a clinical lacunar syndrome^15^ with a compatible lesion on MRI (subcortical infarct ≤15 mm in diameter). All patients underwent full stroke investigation, including brain MRI, imaging of the carotid arteries with ultrasound, computed tomography or MR angiography, and ECG. Echocardiography was performed when clinically indicated (in 54% of all cases). All MRIs and clinical histories were reviewed centrally by one physician (H.S.M.). Exclusion criteria were stenosis >50% in the extra- or intracranial cerebral vessels or previous carotid endarterectomy; cardioembolic source of stroke, defined according to the TOAST (Trial of ORG 10172 in Acute Stroke Treatment) criteria^16^ as high or moderate probability; cortical infarct on MRI; subcortical infarct >15 mm in diameter because these can be caused by embolic mechanisms (striatocapsular infarcts); and any other specific cause of stroke (eg, lupus anticoagulant, cerebral vasculitis, and dissection). All cases were screened for CADASIL (Cerebral Autosomal Dominant Arteriopathy With Subcortical Infarcts and Leukoencephalopathy) and Fabry disease mutations; positive cases were subsequently excluded.^17^

One thousand nine hundred sixty-one unrelated white controls, free of clinical cerebrovascular disease, were obtained by random sampling from general practice lists from the same geographical locations as the patients. Sampling was stratified for age and sex.

All patients and controls underwent a standardized clinical assessment and completed a standardized study questionnaire. MRI was not performed in controls.

### Risk Factors

Data on demographics and risk factors were collected prospectively on a uniform pro forma. A history of cardiovascular risk factors was defined as the presence of these risk factors, either in medical history or when identified during the clinical assessment. Hypertension was defined as on treatment with antihypertensive drugs or systolic blood pressure >140 mm Hg or diastolic blood pressure >90 mm Hg, or both, measured at least 1 week after stroke onset or current treatment with antihypertensive drugs. Diabetes mellitus was defined as on treatment or as at least 2 random venous plasma glucose readings >11.1 mmol/L or 2 consecutive fasting plasma glucose levels >7.0 mmol/L taken after the acute stroke episode. Hypercholesterolemia was defined as on-drug treatment or a serum total cholesterol >5.2 mmol/L. Smoking was defined as smoking at least 1 cigarette per day at any time during life. Excessive alcohol consumption was defined as consuming >200 g of pure alcohol per week. A history of myocardial infarction (MI) and peripheral vascular disease was recorded based on clinical history.

In patients, only blood creatinine was measured and used to estimate glomerular filtration rate, calculated with the chronic kidney disease epidemiology collaboration equation.^18^

### Subtyping of Lacunar Stroke

WMH was graded on MRI using a modified semiquantitative Fazekas scale, which has been shown to reflect pathological severity of small vessel disease in a postmortem validation study.^19^ The Fazekas score was dichotomized in absent or mild degree of WMH (equivalent to Fazekas score <2) and moderate or severe confluent WMH (equivalent to Fazekas grade ≥2) according to a previously validated method.^20^

Lacunes were manually identified using T1-weighted, T2-weighted and fluid-attenuated inversion recovery images. A lacune was defined as a CSF-filled cavity with a diameter of 3 to 15 mm and surrounding rim of fluid-attenuated inversion recovery hyperintensity.^21^

Location of the acute lacunar infarct was classified as deep grey nuclei (basal ganglia and thalamus)/internal capsule, centrum semiovale, and brain stem.

### Statistical Analysis

Approximately 2.1% of the study subjects had missing data on one or more of the assessed risk factors. Missing data in the vascular risk factors were handled using multiple imputation by the method of chained equations,^22,23^ under the assumption of being missing at random. Five complete imputed datasets were created using predictive mean matching and logistic regression for numeric and binary variables, respectively. All variables considered in the subsequent regression analyses were included in the imputation model. Regression analyses were performed on each of the imputed datasets individually, and subsequently the coefficients were pooled using Rubin rules.^24^ Restricting the analyses to only patients with complete data yielded similar point estimates as obtained in the imputed datasets.

The association of single risk factors with patients versus controls and lacunar stroke was assessed using logistic regression analysis, adjusting for age and sex. A test for interaction with age was performed for all single risk factors that were associated with lacunar stroke versus controls. Multivariable logistic regression analysis was used to compare risk factor profiles between lacunar stroke patients and controls. Differences in risk factor profiles in lacunar stroke subtypes versus controls were compared using multinomial regression analysis. Finally, we compared risk factor profiles between lacunar stroke subtypes using multivariable regression analysis. All risk factors, independent of their statistical significance in univariable analysis, were entered in the multivariable models.

Two-sided *P* values <0.05 were considered statistically significant. Statistical analysis was done using the statistical software R version 3.3.1 (http://www.R-project.org).

## Results

### Demographic and Clinical Characteristics

The demographic and clinical characteristics of lacunar stroke patients and controls are shown in Table 1. Lacunar syndrome was pure motor in 37%, pure sensory in 12%, sensorimotor in 26%, ataxic hemiparesis in 8%, clumsy hand/dysarthria in 4%, and atypical lacunar syndrome in 13%. Among lacunar stroke patients without or mild WMH, 515 (74.7%) patients had an isolated infarct, and 174 (25.3%) patients had multiple lacunar infarcts. Among patients in whom confluent WMH was present, 117 (36.6%) had an isolated lacunar infarct, and 203 (63.4%) patients had multiple lacunar infarcts. The demographic and clinical characteristics of lacunar stroke patients stratified by imaging characteristics are shown in Table I in the online-only Data Supplement. Location of the qualifying lacunar infarct was classified as deep grey nuclei/internal capsule in 506 patients and centrum semiovale in 296 patients.

**Table 1.**
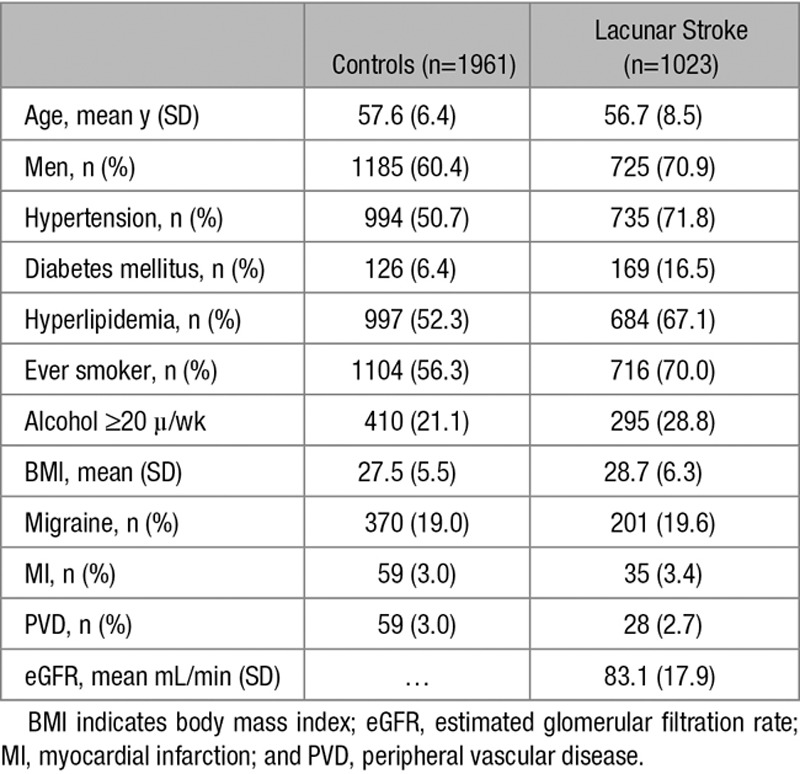
Demographics and Clinical Characteristics of Lacunar Stroke Patients and Controls

### Differences in Risk Factor Profile Between Patients and Controls

Univariable analysis, adjusted for age and sex, showed significant associations with lacunar stroke for hypertension, diabetes mellitus, hyperlipidemia, smoking, excess alcohol consumption, and body mass index (Table 2). The associations for hypertension, diabetes mellitus, hyperlipidemia, and smoking persisted in multivariable logistic regression analysis, including all demographic and clinical characteristics. An interaction with age in the association with lacunar stroke was present for diabetes mellitus (*P*=0.0004). The Figure shows the age-specific associations of risk factors with lacunar stroke versus controls.

**Table 2.**
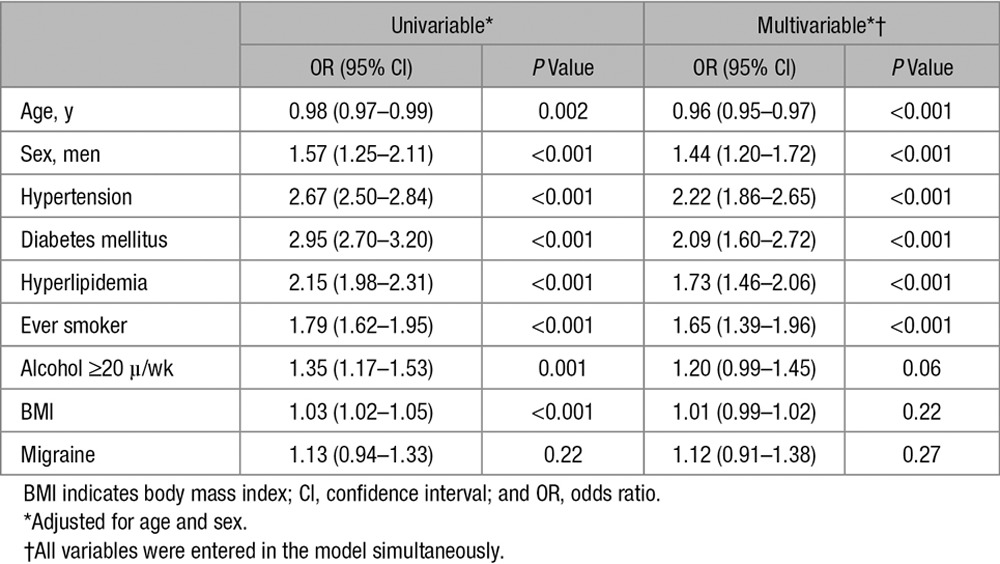
Comparison of Risk Factor Profile Between Patients and Controls

**Figure.**
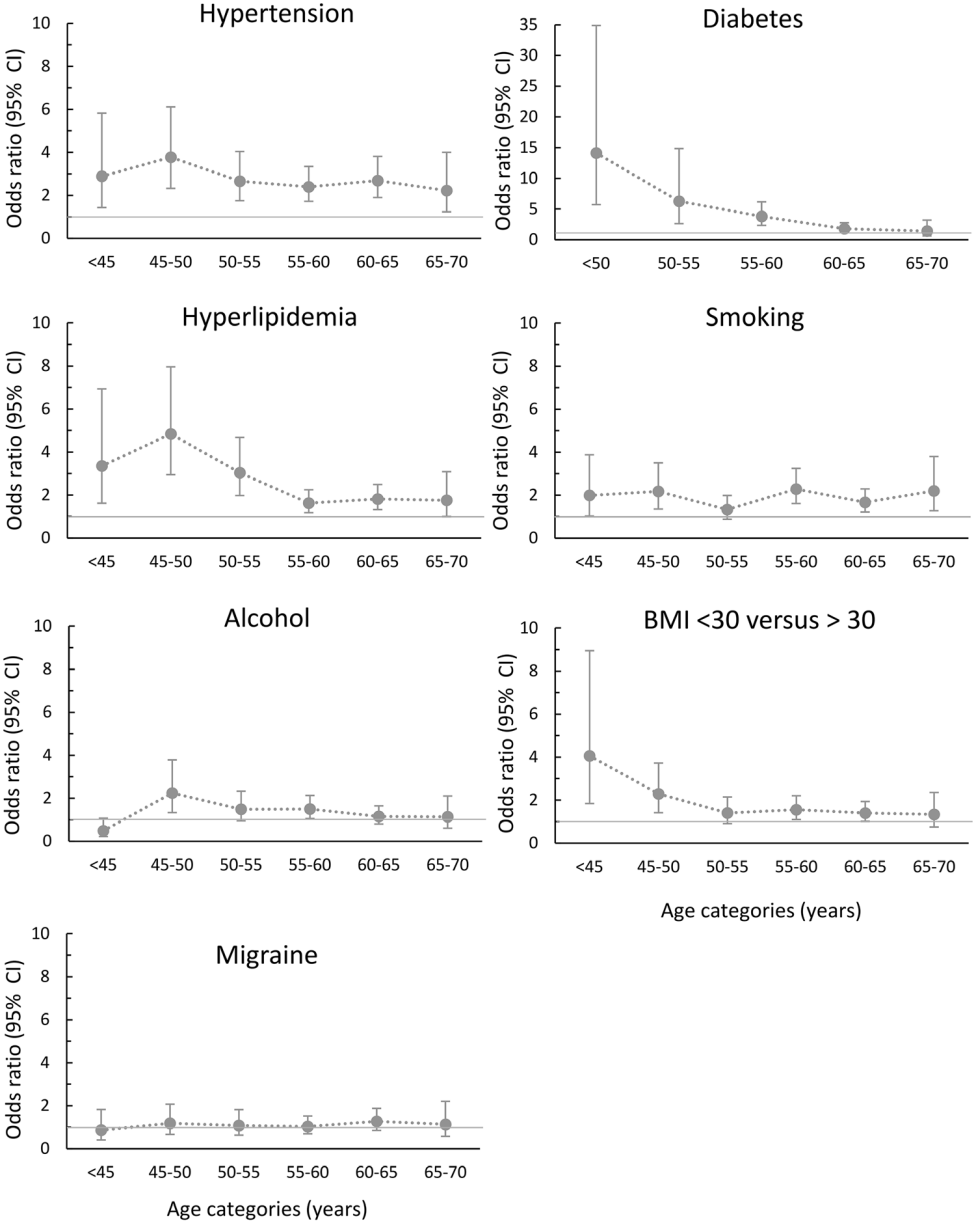
Association of risk factors with lacunar stroke compared with controls according to age category. All odds ratios were adjusted by sex. Bars represent 95% confidence intervals (CIs). BMI indicates body mass index.

### Differences Between Lacunar Stroke Subtypes and Controls

We stratified lacunar stroke patients into 4 groups: no or mild WMH with isolated lacunar infarction (n=515), no or mild WMH with multiple lacunar infarcts (n=116), moderate or severe WMH with isolated lacunar infarction (n=174), or moderate or severe WMH with multiple lacunar infarcts (n=200).

Subsequently, we compared the risk factor profile of lacunar stroke patient subgroups versus controls in multinomial regression. The direction of association of risk factors in cases versus controls were similar across lacunar stroke subgroups except for age (Table 3). Increased age was associated with patients with moderate/severe WMH versus controls, whereas decreased age was associated with no/mild WMH versus controls. Hypertension was associated with all 4 lacunar stroke subgroups versus controls, but this association was especially strong for patients with moderate/severe WMH and multiple lacunar infarcts (odds ratio, 6.75; 95% confidence interval, 4.22–10.79).

**Table 3.**
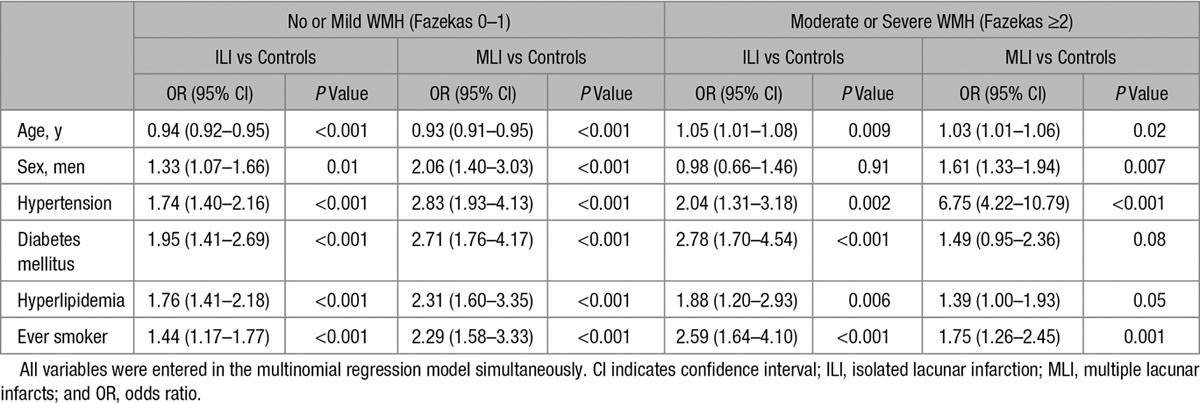
Multivariable Association of Risk Factors in Patients Versus Controls Stratified by Imaging Characteristics

### Differences Between Lacunar Stroke Subtypes

Table 4 shows the results of multivariable logistic regression analyses comparing associations with risk factors between (1) patients with multiple lacunar infarcts versus patients with isolated lacunar infarct, (2) patients with moderate/severe WMH versus no/mild WMH, and (3) patients with an acute infarct in centrum semiovale versus deep grey nuclei/internal capsule. Multiple lacunar infarcts versus isolated lacunar infarcts was associated with male sex and hypertension. Moderate/severe WMH versus no/mild WMH was associated with increased age, hypertension, and decreased estimated glomerular filtration rate.

**Table 4.**
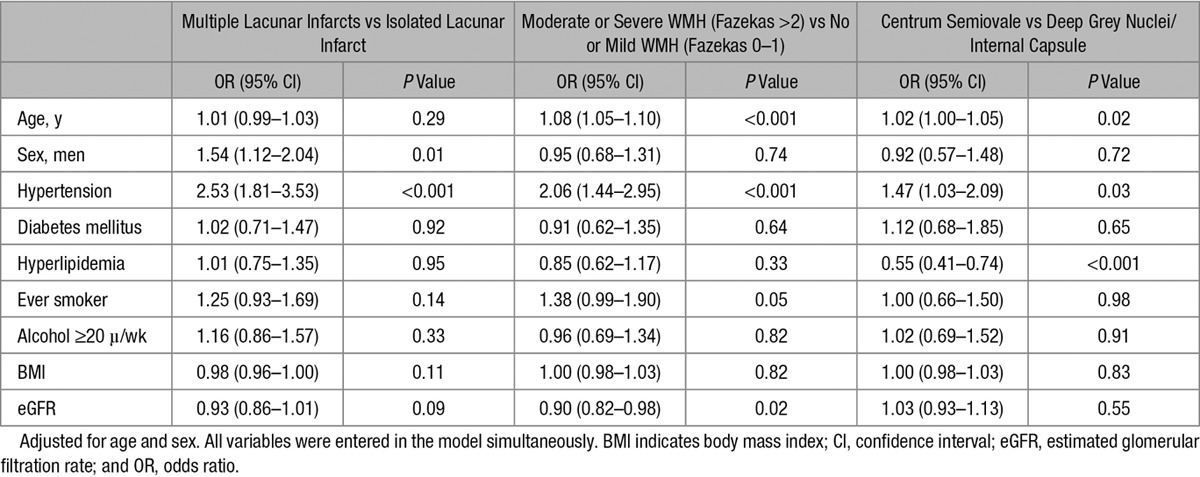
Multivariable Association of Risk Factors in Patients According to Imaging Characteristics

Lacunar infarct in centrum semiovale versus deep grey nuclei/internal capsule was associated with increased age, hypertension, and inversely associated with hyperlipidemia. In addition, independent of other risk factors, centrum semiovale lacunar infarct was associated with multiple lacunar infarcts (odds ratio, 1.64; 95% confidence interval, 1.04–2.59; *P*=0.04) and moderate/severe WMH (odds ratio, 1.62; 95% confidence interval, 1.04–2.59; *P*=0.03).

## Discussion

In this large study of younger-onset MRI-confirmed small vessel disease, we found independent associations with lacunar stroke patients versus controls for hypertension, diabetes mellitus, hyperlipidemia and smoking, and age-specific effects for diabetes mellitus. Risk factor profiles were similar across the lacunar stroke subgroups, but the strength of association with hypertension differed substantially. Patients with a lacunar infarct in the deep grey nuclei/internal capsule are more likely to have hyperlipidemia and younger age, whereas patients with a centrum semiovale infarct are more likely to be older, to have hypertension, and to present with multiple lacunar infarcts and moderate/severe WMH.

Previous studies reported that vascular risk factors, including hypertension, smoking, and diabetes mellitus are at least as common in lacunar stroke as other stroke subtypes.^25^ Also in our study, vascular risk factors were strongly associated with lacunar stroke. The observed age-dependent effect of diabetes mellitus in the risk of lacunar stroke is in line with the findings of the emerging risk factor collaboration—a large meta-analysis of prospective studies on vascular risk factors and vascular disease.^26^ In this study, hazard ratios for ischemic stroke in diabetic people versus nondiabetic people were doubled in those aged 40 to 59 years compared with those ≥70 years.

Boiten et al^11^ first proposed the hypothesis that 2 types of small vessel disease exist; single, symptomatic lacunar infarct in the presence of the usual vascular risk factors and multiple lacunar infarcts with WMH, which was associated with hypertension in most cases. Consistent with this hypothesis, we found distinct risk factor profiles for these 2 radiological subtypes, with the association with hypertension was much stronger in patients with both multiple lacunar infarcts and moderate/severe WMH.

Furthermore, the present study found estimated glomerular filtration rate to be associated with the multiple moderate/severe WMH versus no/mild WMH. The association of renal function with the moderate/severe WMH supports the idea of a potential systemic underlying disease mechanism in this subtype.

Findings of previous studies that compared risk factor profiles between small vessel disease subtypes are summarized in Table 5. The findings of previous studies show much variation, which can be explained at least partly by methodological differences. Previous studies that investigated the risk factor profile of stroke subtypes have been limited by small sample sizes, use of computed tomography instead of MRI, univariable analyses, or inconsistent definitions of risk factors.^27^ Furthermore, there was much variation in the methods used to classify stroke subtypes. The classification methods used were mostly based on clinical symptoms supported by imaging, with some also including risk factors in their definitions of stroke subtype (TOAST classification^16^); for example, including history of hypertension as a criteria for lacunar stroke will artificially inflate any association between lacunar stroke and hypertension.

**Table 5.**
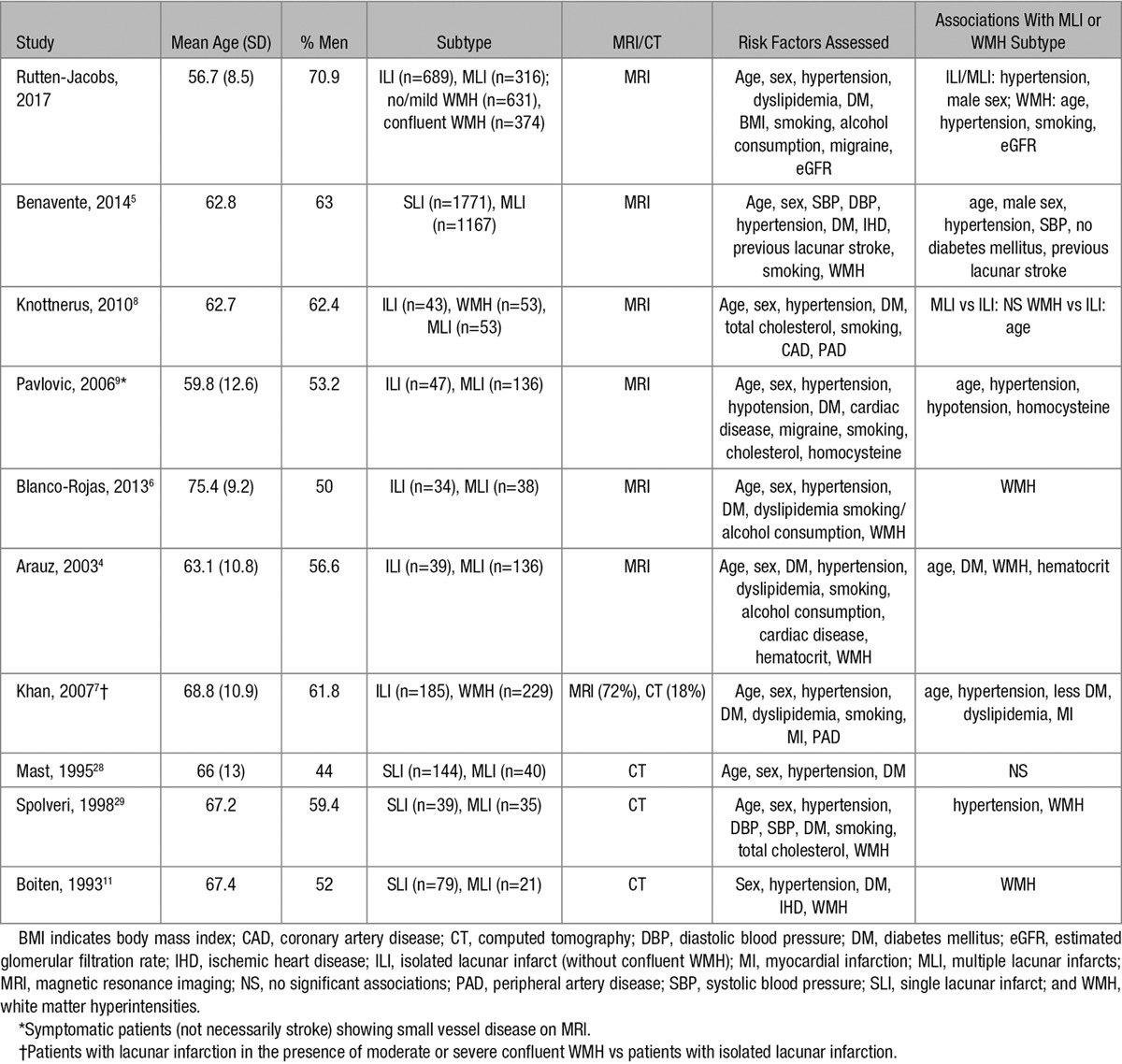
Studies on Risk Factor Associations With Small Vessel Disease Subdivisions

In the present study, infarcts in the deep grey nuclei/internal capsule were more often isolated lacunar infarcts and were associated with hyperlipidemia, whereas centrum semiovale infarcts were associated with hypertension and the presence of multiple lacunar infarcts and WMH. These findings might indicate that the previously suggested subtypes of lacunar stroke differ according to location of the acute infarct (deep grey nuclei/internal capsule versus centrum semiovale).^11^

There are few previous studies that investigated differences in risk factors according to location of lacunar infarct, and these studies show considerable variation in categorization of location. A previous article that took a similar approach to the current study, comparing centrum semiovale lacunar infarcts versus deep grey nuclei/internal capsule, did not demonstrate the association of hypertension with centrum semiovale and hyperlipidemia with deep grey nuclei/internal capsule.^13^ However, the sample size of this study was only a quarter from that in the present study.

Strengths of our study include the large sample size and prospective inclusion of the patients and controls in multiple centers using an identical protocol and standardized forms across all centers. Another important strength is that lacunar stroke was confirmed in all patients using MRI and that all scans were rated by one single rater. Reliability of the grading was high because regrading 20 randomly selected scans showed perfect agreement of regrading lacunar stroke subtype. In addition, we included a relatively young population with a mean age of only 57 years, avoiding substantial heterogeneity because of possible other pathologies at older age.

Our study had also some limitations. MRI scanning was not included in the study protocol for controls because of logistical reasons but also to avoid a decrease in participation rate, which could lead to selection bias among controls. However, controls did not have symptomatic cerebral vascular disease because this was an exclusion criterion.

Furthermore, the risk factor profile that we investigated in the present study only included classical vascular risk factors. Future studies are needed that examine the role of emerging risk factors like inflammation, genetics, behavioural factors, and environmental factors.

In summary, in this large population of younger-onset MRI-confirmed lacunar stroke, we describe the distinctive vascular risk factor profile of lacunar stroke patients. Furthermore, our data provide evidence for differing subtypes of lacunar stroke with distinct risk factor profiles, supporting the hypothesis that these radiological subtypes have different underlying pathophysiology.

## Appendix

Center-specific collaborators are reported in the online-only Data Supplement.

## Acknowledgments

We thank all study staff and participants for their important contributions. Detailed acknowledgments are reported in the online-only Data Supplement.

## Sources of Funding

Dr Rutten-Jacobs was supported by a British Heart Foundation Immediate Research Fellowship (FS/15/61/31626). The UK young lacunar stroke DNA study was funded by a grant from the Wellcome Trust (WT072952) and the Stroke Association (TSA 2010/01). The sponsors of the study had no role in the study design, data collection, data analysis, interpretation, writing of the article, or the decision to submit the article for publication. H.S. Markus was supported by a National Institute for Health Research Senior Investigator award and is supported by the Cambridge University Trust National Institute for Health Research Biomedical Research Centre.

## Disclosures

None.

## Supplementary Material
