# Supplementary material for: Vascular Risk Factor Profiles Differ Between Magnetic Resonance Imaging-Defined Subtypes of Younger-Onset Lacunar Stroke
Source: Stroke. 2017 Aug 1;48(9):2405–11. doi: 10.1161/STROKEAHA.117.017813 (PMC5571884; doi:10.1161/STROKEAHA.117.017813)
Supplement: Supplementary file 1 [file str-48-2405-s001.pdf]

SUPPLEMENTAL MATERIAL

Vascular Risk Factor Profiles Differ Between MRI Defined Subtypes of Younger Onset Lacunar Stroke

Loes C.A. Rutten-Jacobs, PhD[1], Hugh S Markus, FRCP[1], on behalf of the UK YOUNG LACUNAR STROKE DNA STUDY

Affiliations
[1] Stroke Research Group, Department of Clinical Neurosciences, University of Cambridge, Cambridge, UK

Corresponding Author:
Loes Rutten-Jacobs
Department of Clinical Neurosciences
University of Cambridge
CB2 0QQ Cambridge, UK
+44 (0)1223 217697 (Phone)
LR406@medschl.cam.ac.uk

Supplemental Table I Demographics and clinical characteristics of lacunar stroke patients stratified by imaging characteristics.

	No or mild WMH (Fazekas 0-1)		Moderate or severe WMH (Fazekas > 2)	
	ILI n = 515	MLI n = 174	ILI n = 117	MLI n = 203
Age, mean years (SD)	54.9 (9.0)	55.1 (7.9)	60.4 (7.1)	59.9 (6.9)
Male, n (%)	356 (69.1)	137 (78.7)	71 (61.2)	148 (74.0)
Hypertension, n (%)	324 (62.9)	132 (75.9)	86 (74.1)	179 (89.5)
Diabetes, n (%)	74 (14.4)	38 (21.8)	26 (22.4)	28 (14.0)
Hyperlipidaemia, n (%)	331 (64.3)	125 (71.8)	86 (74.1)	130 (65.0)
Ever smoker, n (%)	336 (65.2)	132 (75.9)	91 (78.4)	145 (72.5)
Alcohol ≥ 20 u/wk	145 (28.2)	58 (33.3)	25 (21.6)	61 (30.5)
BMI, mean (SD)	28.8 (6.4)	28.3 (5.7)	29.1 (6.7)	28.3 (5.9)
Migraine, n (%)	105 (20.4)	30 (17.2)	24 (20.7)	42 (21.0)
MI, n (%)	12 (2.3)	6 (3.4)	7 (6.0)	8 (4.0)
PVD, n (%)	9 (1.7)	1 (0.6)	4 (3.4)	14 (7.0)
eGFR, mean ml/min (SD)	85.8 (16.7)	83.4 (18.3)	77.6 (18.0)	79.0 (19.1)

Abbreviations: ILI, isolated lacunar infarction; MLI, multiple lacunar infarcts; SD, standard deviation; BMI, body mass index, MI, myocardial infarction; PVD, peripheral vascular disease; eGFR, estimated glomerular filtration rate

Detailed acknowledgements

UK Young Lacunar Stroke DNA Study collaborators

Study managers: Josie Monaghan; Alan Zanich, Samantha Febrey, Eithne Smith, Jenny Lennon, St George's University of London

Participating centres (number of enrolled patients per centre; local investigators): Aberdeen Royal Infirmary, Aberdeen (12; Mary Macleod). Addenbrooke's Hospital, Cambridge (54; Jean-Claude Baron, Elizabeth Warburton, Diana J Day, Julie White). Airedale General Hospital, Steeton (4; Samantha Mawer). Barnsley Hospital, Barnsley (3; Mohammad Albazzaz, Pravin Torane, Keith Elliott, Kay Hawley). Bart's and the London, London (2; Patrick Gompertz). Basingstoke and North Hampshire Hospital, Basingstoke (13; Elio Giallombardo, Deborah Dellafera). Blackpool Victoria Hospital, Blackpool (11; Mark O'Donnell). Bradford Royal Infirmary, Bradford (1; Chris Patterson). Bristol Royal Infirmary, Bristol (8; Sarah Caine). Charing Cross Hospital, London (12; Pankaj Sharma). Cheltenham General and Gloucester Royal Hospitals, Cheltenham and Gloucester (10; Dipankar Dutta). Chesterfield Royal Hospital, Chesterfield (4; Sunil Punnoose, Mahmud Sajid). Countess of Chester Hospital, Chester (22; Kausik Chatterjee). Derriford Hospital, Plymouth (4; Azlisham Mohd Nor). Dorset County Hospital NHS Foundation Trust, Dorchester (6; Rob Williams). East Kent Hospitals University NHS Foundation Trust, Kent (22; Hardeep Baht, Guna Gunathilagan). Eastbourne District General Hospital, Eastbourne (4; Conrad Athulathmudali). Frenchay Hospital, Bristol (1; Neil Baldwin). Frimley Park Hospital NHS Foundation Trust, Frimley (6; Brian Clarke). Guy's and St Thomas' Hospital, London (14; Tony Rudd). Institute of Neurology, London (25; Martin Brown). James Paget University Hospital, Great Yarmouth (1; Peter Harrison). King's College Hospital, London (16; Lalit Kalra). Leeds Teaching Hospitals NHS Trust, London (125; Ahamad Hassan). Leicester General Hospital and Royal Infirmary, Leicester (9; Tom Robinson, Amit Mistri). Luton and Dunstable NHSFT University Hospital, Luton (16; Lakshmanan Sekaran, Sakthivel Sethuraman, Frances Justin). Maidstone andTunbridge Wells NHS Trust (3; Peter Maskell). Mayday University Hospital, Croydon (14; Enas Lawrence). Medway Maritime Hospital, Gillingham (5; Sam Sanmuganathan). Milton Keynes Hospital, Milton Keynes (1; Yaw Duodu). Musgrove Park Hospital, Taunton (9; Malik Hussain). Newcastle Hospitals NHS Foundation Trust, Newcastle upon Tyne (12; Gary Ford). Ninewells Hospital, Dundee (5; Ronald MacWalter). North Devon District Hospital, Barnstaple (8; Mervyn Dent). Nottingham University Hospitals, Nottingham (17; Philip Bath, Fiona Hammonds). Perth Royal Infirmary, Perth (2; Stuart Johnston). Peterborough City Hospital, Peterborough (1; Peter Owusu-Agyei). Queen Elizabeth Hospital, Gateshead (5; Tim Cassidy, Maria Bokhari). Radcliffe Infirmary, Oxford (5; Peter Rothwell). Rochdale Infirmary, Rochdale (4; Robert Namushi). Rotherham General Hospital, Rotherham (1; James Okwera). Royal Cornwall Hospitals NHS Trust, Truro (11; Frances Harrington, Gillian Courtauld). Royal Devon and Exeter Hospital, Exeter (22; Martin James). Royal Hallamshire Hospital, Sheffield (1; Graham Venables). Royal Liverpool University Hospital and Broadgreen Hospital, Liverpool (9; Aravind Manoj). Royal Preston Hospital, Preston (18; Shuja Punekar). Royal Surrey County Hospital, Guildford (23; Adrian Blight, Kath Pasco). Royal Sussex County Hospital, Brighton (14; Chakravarthi Rajkumar, Joanna Breeds). Royal United Hospital, Bath (6; Louise Shaw, Barbara Madigan). Salford Royal Hospital, Salford (16; Jane Molloy).

Southampton General Hospital, Southampton (1; Giles Durward). Southend Hospital, Westcliff-on-Sea (26; Paul Guyler). Southern General Hospital, Glasgow (34; Keith Muir, Wilma Smith). St George's Hospital, London (108; Hugh Markus, Ahamad Hassan). St Helier Hospital, Carshalton (10; Val Jones). Stepping Hill Hospital, Stockport (4; Shivakumar Krishnamoorthy). Sunderland Royal Hospital, Sunderland (1; Nikhil Majumdar). The Royal Bournemouth Hospital, Bournemouth (15; Damian Jenkinson). The Walton Centre, Liverpool (15; Richard White). Torbay Hospital, Torquay (19; Debs Kelly). University Hospital Aintree, Liverpool (19; Ramesh Durairaj). University Hospital of North Staffordshire, Stoke-on-trent (16; David Wilcock). Wansbeck General Hospital and North Tyneside Hospital, Ashington and North Shields (6; Christopher Price). West Cumberland Hospital, Whitehaven (6; Olu Orugun, Rachel Glover). West Hertfordshire Hospital, Watford (20; David Collas). Western General Hospital, Edinburgh (12; Cathie Sudlow). Western Infirmary, Glasgow (33; Kennedy R. Lees, Jesse Dawson). Wycombe Hospital and Stoke Mandeville, High Wycombe (20; Dennis Briley and Matthew Burn). Yeovil District Hospital, Yeovil (46; Khalid Rashed). York Teaching Hospital, York (1; John Coyle).

Controls coordination centres (local investigator): Aberdeen Royal Infirmary, Aberdeen (Mary Macleod). Addenbrooke's Hospital, Cambridge (Jean-Claude Baron). Nottingham University Hospitals, Nottingham (Philip Bath). St George's Hospital, London (Hugh Markus).
